# Impact of Biogenic Amines on Food Quality and Safety

**DOI:** 10.3390/foods8020062

**Published:** 2019-02-08

**Authors:** Claudia Ruiz-Capillas, Ana M. Herrero

**Affiliations:** Department of Products, Institute of Food Science, Technology and Nutrition, ICTAN-CSIC, Ciudad Universitaria, 28040 Madrid, Spain; ana.herrero@ictan.csic.es

**Keywords:** biogenic amines, food products, food quality, food safety, quality control, quality indexes, public health, legislation–regulation, analytical determination

## Abstract

Today, food safety and quality are some of the main concerns of consumer and health agencies around the world. Our current lifestyle and market globalization have led to an increase in the number of people affected by food poisoning. Foodborne illness and food poisoning have different origins (bacteria, virus, parasites, mold, contaminants, etc.), and some cases of food poisoning can be traced back to chemical and natural toxins. One of the toxins targeted by the Food and Drug Administration (FDA) and European Food Safety Authority (EFSA) is the biogenic amine histamine. Biogenic amines (BAs) in food constitute a potential public health concern due to their physiological and toxicological effects. The consumption of foods containing high concentrations of biogenic amines has been associated with health hazards. In recent years there has been an increase in the number of food poisoning cases associated with BAs in food, mainly in relation to histamines in fish. We need to gain a better understanding of the origin of foodborne disease and how to control it if we expect to keep people from getting ill. Biogenic amines are found in varying concentrations in a wide range of foods (fish, cheese, meat, wine, beer, vegetables, etc.), and BA formation is influenced by different factors associated with the raw material making up food products, microorganisms, processing, and conservation conditions. Moreover, BAs are thermostable. Biogenic amines also play an important role as indicators of food quality and/or acceptability. Hence, BAs need to be controlled in order to ensure high levels of food quality and safety. All of these aspects will be addressed in this review.

## 1. Biogenic Amines and Food Safety

Food safety is one of the main concerns of consumer and health agencies around the globe (European Food Safety Authority (EFSA), Food and Drug Administration (FDA), Food Safety Commission of Japan (FSCJ), World Health Organization (WHO), etc.). According to the WHO, more than 200 diseases are transmitted by food and the vast majority of the population will contract a foodborne disease at some point in their lifetime. For example, in the U.S. 48 million people (one in six) suffer a foodborne disease each year. Of these, 128,000 are hospitalized and 3000 die from such diseases [1,2]. Moreover, the real numbers are higher as many cases of foodborne disease go undetected and are not recorded as such, due to the difficulty in establishing a causal relationship between food contamination and illness or death. This highlights the importance of making sure that the food we consume is not contaminated with potentially harmful elements at any point along the food chain. Because food can become contaminated at any point along the global supply chain during production, distribution, and preparation–consumption, each individual along this chain, from producer to consumer, has a role to play in ensuring that the food we eat does not cause disease. Furthermore, if we are to prevent such disease, we must gain a deeper understanding of the origin of foodborne illness and the way to control it.

The origin of foodborne illness could be bacteria, virus, parasite, mold, contaminants, metals, allergens, pesticides, natural toxins, etc., that can contaminate food and cause disease. In general, most food poisoning is caused by bacteria, viruses, and parasites as opposed to toxic substances. Nonetheless, there are cases of food poisoning that can be linked to chemical or natural toxins. From among these toxins, the FDA and EFSA pay particular attention to aflatoxins, mycotoxins, histamine, etc. Of these, it is worth noting that histamine, a biogenic amine, is present in most foods but in greater abundance in fish and fishery products. This biogenic amine is the main component in “scombrid poisoning” or “histamine poisoning” since these intoxications are related to the consumption of fish of the *Scombridae* and *Scomberesocidae* families (tuna, mackerel, bonito, bluefish, etc.) containing high levels of histamine. These species contain high levels of the free amino acid histidine in their muscle tissue, which is decarboxylated to histamine. However, other non scombroid species also contain high levels of free histamine in their muscle tissue [3,4], which is why this illness came to be known as “histamine poisoning”. There have been recent cases involving vacuum-packed salmon. The most common symptoms of histamine poisoning are due to the effects it has on different systems (cardiovascular, gastrointestinal, respiratory, etc.) producing low blood pressure, skin irritation, headaches, edemas, and rashes typical of allergic reactions [5,6]. Furthermore, histamine plays a role in the health problem known as histaminosis or histamine intolerance associated with the increase of histamine in plasma [4]. It is also important to point out that histamine is a mediator of allergic disorders. Biogenic amines are released by mast cell degranulation (in response to an allergic reaction) and the consumption of foods containing histamine can have the same effect. Since food allergy symptoms are similar to those of histamine poisoning (food intolerance), physicians occasionally make a faulty diagnosis. For all these reasons, histamine is the biogenic amine (BA) causing major concerns in clinical and food chemistry. However, we would note that apparently histamine is not the only agent causing scombroid poisoning [7,8,9,10,11,12]. Other amines, such as putrescine and cadaverine, are also associated with this illness, although both seem to have much lower pharmacological activity on their own but enhance the toxicity of histamine and decrease the catabolism of this amine when they interact with amine oxidases, thus favoring intestinal absorption and hindering histamine detoxification [13,14].

Another important biogenic amine related to food poisoning is tyramine. In this case, intoxication is known as the “cheese reaction” as it is associated with the consumption of foods with high concentrations of tyramine, mainly associated with the consumption of cheese [10,14,15,16,17]. However, high levels of tyramine have also been observed in meat and meat products [14,18,19,20,21]. As in the case of histamine, this illness came to be known as “tyramine reaction” because of the main compound involved. Typical symptoms of tyramine poisoning are migraines, headaches, and increased blood pressure, since tyramine sparks the release of noradrenaline from the sympathetic nervous system [5,6,10].

Other Bas, such as spermidine or spermine, have also been associated with food allergies [6,22,23]. Tyramine and β-phenylethylamine are suspected of triggering hypertensive crises in certain patients and of producing dietary-induced migraines. Although tryptamine has toxic effects on humans (causing blood pressure to increase, thus leading to hypertension), the maximum amount of tryptamine permitted in sausages is not regulated in some countries [23]. It is worth noting an additional toxicological risk associated with BAs, mainly secondary BAs (putrescine and cadaverine), which are involved in other kinds of food poisoning, such as the formation of nitrosamines, that are believed to be cancer causing compounds [24,25]. This risk is greatest in meat products with high biogenic amine levels and which contain nitrite and nitrate salts used as curing agents, and also with heat treated products, as these factors favor interaction between BAs and nitrites to form nitrosamines [25,26]. However, under normal circumstances, the human body possesses detoxification systems to take care of these BAs, mainly in the intestine through the action of monoamine oxidase (MAO; CE 1.4.3.4), diamine oxidase (DAO; CE 1.4.3.6), and polyamine oxidase (PAO; CE 1.5.3.11). However, in certain cases this mechanism can be hindered by a variety of factors or circumstances, and BAs could accumulate in the body and cause serious toxicological problems and a high risk of poisoning [4,14]. Factors that could alter the detoxification mechanism include the consumption of amine oxidase inhibitors (mono and diamine oxidase inhibitors (MAOI/DAOI)), alcohol, immune deficiency of the consumer, gastrointestinal disorders, large amounts of BA, for example in the case of spoiled or fermented foods, etc. [5,13,27]. When calculating BA intake, one must consider that foods are not typically consumed in isolation but rather in the context of a meal where several foods are eaten simultaneously (meat, fish, cheese, wine, vegetables, etc.). Therefore, the aggregate amount of BA consumed would be the sum of all the amines from the different foods rather than one food considered individually. The potential toxicological effect would be the sum of the amines in all of the different foods, the synergies between them, and the other personal factors mentioned above. The role of various substances that enhance the toxicity of BA and the existence of synergic effects have been demonstrated. For example, in Europe approximately 20% of the population regularly takes MAOI and/or DAOI antidepressant drugs. In such circumstances, not even low amounts of biogenic amines can be metabolized efficiently, the result being increased sensitivity to BAs [14]. Some authors [28,29] have suggested that ripened meat products (“chorizo”, “salchichón”, “salami”, etc.) contain enough tyramine to poison people taking MAOI even with low levels of tyramine (in the 6–9 mg/kg range). The consumption of 100 g of any of these products would interact with MAOI, while in the absence of MAOI none of these processed meats would be toxic if ingested in normal amounts, always depending of course on individual susceptibility. A new generation of MAOI has been developed that diminishes this sensitivity. The ingestion of even small amounts of tyramine has been known to cause severe migraines with intracranial hemorrhaging in patients treated with classic MAOIs, while tyramine between 50 and 150 mg is better tolerated by patients treated with a new generation of MAOIs, i.e., the so-called RIMA (reversible MAO-A inhibitor) [4,30]. The market is currently offering pharmaceutical preparations based on the DAO enzyme for the treatment of migraines whose fundamental function is to mitigate deficiencies of this enzyme (DAO), thus favoring the metabolism of histamine. It is very difficult to establish toxicity parameters for BAs considering the number of factors that affect their toxicity.

## 2. Biogenic Amines and Quality Control of Food Products

It is important to control and monitor biogenic amines not only for toxicological and health reasons as mentioned above, but also because they may play an important role as quality and/or acceptability indicators in some foods, and managing this quality is also a way to guarantee and ensure food safety. Food quality refers to main characteristics having to do with safety, nutrition, availability, convenience, integrity, and freshness [31].

BAs have been frequently employed as quality indexes in various foods (meat, fish, wines, etc.) to signal their degree of freshness and/or deterioration and also to control the processing and development of food and beverages. Individual BAs, such as histamine, tyramine, cadaverine, or a combination of various amines (putrescine–cadaverine, spermidine–spermine, etc.), have likewise been used as a quality index [19,26,32,33,34,35,36,37,38,39,40]. Also, different BA-based quality indexes have been proposed, such as the traditional one developed by Miet and Karmas [32] used as an indicator of the decomposition of fish. This index is based on the increase in putrescine, cadaverine, and histamine levels and the decrease in spermidine and spermine levels throughout the fish storage process. Scores of 0 and 1 are indicative of good quality fish, between 1 and 10 are tolerable, and a score of over 10 indicates decomposition of the product. However, in the case of other foods, such as cheese, meat, and meat products, this index has not yielded good results mainly because it does not include levels of tyramine, the main biogenic amine in these products. An alternative biogenic amine index (BAI) has been proposed for meat that consists of the sum of putrescine, cadaverine, histamine, and tyramine [40,41]. Hernández-Jover et al. [41] also suggested quality ranges for the index: BAI <5 mg/kg indicating good quality fresh meat, between 5 and 20 mg/kg for acceptable meat but with signs of initial spoilage, between 20 and 50 mg/kg for low quality meat, and >50 mg/kg for spoiled meat. However, the usefulness of BAs as a quality index depends on many factors, mainly concerning the nature of the product (fresh, canned, modified atmosphere, fermented, etc.). For example, BA indexes have proven to be more satisfactory in fresh meat and meat products and heat-treated products than in fermented products [40]. This is at least partly because biogenic amine concentrations vary much more in fermented products than in fresh and cooked meat products owing to the number of different factors involved in their processing (ripening, maturation, starter, additives, etc.) [13,14,20,21,42,43,44]. Therefore, establishing a biogenic amine index that reliably predicts product quality is no simple matter. It is important to note that sometimes foods with toxic levels of BAs, such as histamine or tyramine often appear organoleptically “normal”. This could be the case of tuna, salmon, or fermented chorizo where unacceptable and toxic levels of histamine are undetectable prior to consumption and therefore consumers are unable to reject products based on sensorial parameters. This is another important reason to control these compounds.

## 3. Biogenic Amines in Food

**Figure 1:**
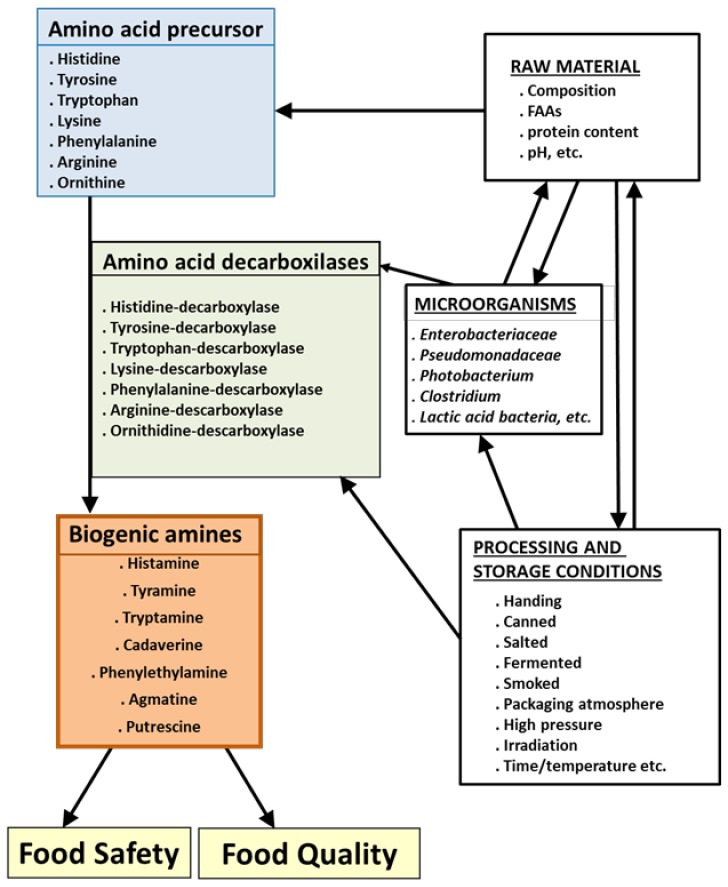
Formation of biogenic amines and factors influencing their formation. FAAs are free amino acids.

Biogenic amines are compounds that are commonly found in food and beverages such as meat, fish, cheese, vegetables, wine, etc. The most important BAs found in food are histamine, tyramine, putrescine, cadaverine, β-phenylethylamine, agmatine, tryptamine, serotonin (SRT), spermidine, and spermine. These dietary amines are classified according to their chemical structure as aromatic amines (histamine, tyramine, serotonin, phenylethylamine, and tryptamine), aliphatic diamines (putrescine and cadaverine), and aliphatic polyamines (agmatine, spermidine, and spermine) [33,45]. In terms of origin or synthesis, they are classified as polyamines when they are endogenous and formed naturally by animals, plants, and microorganisms, which play an important role in physiological functions (neurotransmitter, psychoactive, vasoactive, regulating gene expression, cell growth and differentiation, gastric secretions, immune response, inflammatory processes, etc.), and biogenic amines, when formed mainly by the decarboxylation of free amino acids (FAAs) from the action of decarboxylase enzymes, which are mainly of microbial origin (Figure 1).

BA formation is influenced by numerous factors (Figure 1) that can be divided into three groups: raw materials (composition, pH, ion strength, etc.), microorganisms (decarboxylase activity is attributed chiefly to *Enterobacteriaceae*, *Pseudomonadaceae*, *Micrococcaceae*, lactic acid bacteria, etc.), and processing and storage conditions (fresh, cured, fermented, refrigerated, modified atmosphere, etc.) [9,14,17,43,46,47,48]. These factors do not act in isolation but rather have combined effects that determine the final concentration of BAs in food. Therefore, to ensure food quality from the perspective of BAs, it is vital to use suitable raw materials to limit the presence of BAs in the end product and hence assure better quality. It should be noted that these BAs are thermostable. In other words, once these biogenic amines are produced they are very difficult to destroy by subsequent processing (pasteurization, cooking, etc.) meaning that if they are present in the raw material or product, they will still be present in the final product.

In the case of factors such as microorganisms, it is necessary to control not only the microbial load in the product but also the type of microbiota constituting that load (bacterial species and strain), that in turn depends on factors associated with the raw material and processing and storage conditions [40]. These conditions directly or indirectly affect substrate and enzyme concentrations and determine the presence of other compounds or conditions that modulate (favor or not) decarboxylase activity (pH, temperature, co-factors, etc.). Therefore, there are many factors to be considered, especially in connection with the technology applied (thermal treatments, additives, fermentation, refrigeration, packaging, etc.). Hence, suitable raw materials are not enough to limit BA formation. Processing conditions must also be optimized as they are responsible for the specific profile of the biogenic amine in the different products. For example, fermentation generally promotes BAs, and in fact, this is the group of meat products with the greatest amount and diversity of these compounds. This has to do with several factors, such as the raw material, temperature of the medium (assuring conditions favorable to starter growth), the presence and concentration of additives (sugar, salt, antimicrobial agents, etc.), the microorganisms present, etc. The large quantities of microorganisms in these products, accompanied by proteolysis, gives rise to high concentrations of the amino acids constituting the nutrients required by the bacteria and the substrate on which decarboxylase enzymes work. In some cases, the presence of BAs in fermented products has been attributed to the poor quality of raw materials and defective processing.

The storage temperature of final products is also one of the critical factors in the formation of BAs. Freezing temperatures inhibit microbial growth and therefore the production of biogenic amines. In contrast, higher chilled storage temperatures (>5 °C in fresh meat or fish) or poor temperature control foster the growth of microorganisms in products, which results in an increase in proteolysis in muscle tissue and an increase in decarboxylase enzymes and activity. Hence, low storage temperatures can make for improved quality and longer shelf-life of products. However, an increase in BAs is also related to processing and packaging conditions (modified atmosphere, vacuum, high hydrostatic pressure, irradiation, cooking products, etc.) that have an important influence on microbial flora. Controlling all of these factors improves the quality and shelf-life of food [14,34,48,49,50]. Today’s lifestyle and global markets have led to the massive consumption of food and with this the development of new production and conservation systems and a complex food chain, that in many cases requires a deeper knowledge of how these foods are handled and forces us to face new challenges and problems in supplying safe foods.

## 4. Legislation Concerning Biogenic Amines in Food and Beverages

While it is very difficult to establish BA toxicity ranges owing to the many factors involved as described in the foregoing, given the dual importance of BAs (quality and health implications), efforts are being made to control BAs in food products and all countries have enacted legislation in this respect [4]. However, specific legislation only covers histamine in fishery products and no criteria have been established for other BAs or other food products, such as meat, dairy, or other products, despite the presence of important levels of BA in all types of food and the potential health risk in certain sectors of society where these products are consumed. However, in general the same legislation applicable to fish is applied to these products [19,22,40,44,51,52]. European Commission Regulations (2073/2005, 144/2007, 365/2010) set food safety criteria for histamine in fish. This legislation applies to particular fish species within the *Scombridae*, *Clupeidae*, *Eugraulidae*, *Coryphenidae*, *Pomatomidae*, and *Scomberesocidae* families throughout their shelf life with a sampling plan comprising nine units, two of which may be between 100–200 mg/kg of histamine and none above the limit of 200 mg/kg. This legislation also covers histamine levels in the processing (brine, enzyme maturation, curing, etc.) of these species with a sampling plan comprising nine units, two of which may be between 200–400 mg/kg of histamine and none above the limit of 400 mg/kg. The Australian and New Zealand standard codex feature similar levels between 100 mg/kg and none may exceed the limit of 200 mg/kg. In the U.S. the Food and Drug Administration [3] has set histamine limits in food in general at 50 mg/kg. This legislation is more advanced than its counterpart in the EU insofar as it applies to all food products.

Notwithstanding the difficulties and limitations in determining the real risk of toxicity for consumers posed by BAs in food, we should be aware that this legislation has its limitations. It is designed for one single biogenic amine (histamine) that, while admittedly one of the most important amines from a toxicological point of view, is not the only cause of toxicity. Limits should also be established for other amines, particularly tyramine, that have toxic effects, while also bearing in mind the other factors contributing to toxicity such as toxicity enhancers (individual susceptibility, consumption of MAOI, synergies resulting from the consumption of different foods during the same meal, etc.), with a view to establishing more restrictive legislation in certain cases. Although these aspects are truly difficult to address, they should be studied and included in future regulations to guarantee food safety and consumer health.

## 5. Analytical Determination of Biogenic Amines

As noted above, from the point of view of food safety and to assess the potential toxic effect of BAs, it is important to control and determine which BAs should be addressed. A number of swift and accurate analytical methods have been developed to determine BA levels in different foods and they were collected in various reviews [36,53,54,55,56,57,58]. These methods range from the more traditional colorimetric and fluorometric methods focused mainly on determining histamine individually, as is also the case with fast commercial kits based on the Elisa enzyme immunoassay to detect histamine in fish, to methods allowing for the simultaneous determination of several BAs (preferable) using chromatography methods such as: gas chromatography (CG) and gas chromatographic–mass spectrometry, high-performance liquid chromatography (HPLC), HPLC-tandem mass spectrometry, flow injection analysis (FIA), capillary electrophoresis, etc. (Table 1). Of all of these methods, HPLC is the most popular and frequently reported for the separation and quantification of BAs. This is the specific analytical method in European Commission (EC) [4]. This procedure offers high resolution, sensitivity, and versatility, and sample treatments are generally simple. Moreover, it offers the advantage of analyzing several BAs simultaneously. The HPLC method involves the first phase of BA extraction from the products and a second phase of determination. The extraction of BA is conducted using different solvents, such as hydrochloric acid, trichloroacetic acid, perchloric acid, methanol, etc., for the extraction procedure depending on the type of matrix (Table 1). The complexity of these matrices is a critical consideration for the adequate recovery of all BAs and to prevent interference with other compounds in the samples. This phase is also necessary in many other methods (Table 1). Chromatographic determination by HPLC is generally used pre- and post-column with reverse phase or ion exchange columns. Depending on the type of column employed, different derivate reagents are used to increase the sensitivity of the determination since BAs have low volatility and lack chromophores. The reagents commonly used in the literature are: dansyl chloride, ortho-ophtaldehyde (OPA), benzoyl chloride, p-phenyldiazonium sulfonate, 3-(4-fluorobenzoyl)-2-quinolinecarboxaldehyde, methanesulfonic acid, etc. Of these, OPA and dansyl chloride are the ones most widely used. The type of derivatization reagent used has implications for detection systems: UV/Vis, diode array, and fluorescence detector (Table 1). Important advances in analytical methods have paved the way for the use of more routine methods, such as flow injection analysis (FIA), which has been successfully used to determine BAs. This methodology offers a number of advantages, such as easy control of the chemical reaction, rapid reaction in the system, all reagent additions are performed automatically, etc. Moreover, FIA methods have been extensively used in combination with mass spectroscopy and with immobilized enzymes and electrodes or reactors using several different enzymes (amine oxidase, peroxidase, histaminase, etc.) to determine BAs in various elements by means of amperiometry or chemiluminescence. This has marked a major step forward in biosensor-assisted FIA determination of BAs [53].

## 6. Conclusions

There are many reasons to prevent the accumulation of biogenic amines in food products, mainly related to their utility as food quality indicators and their potential implications for consumer health. Controlling these compounds implies a deep understanding of the formation, monitoring, and reduction of biogenic amines during the processing and storage of food, and even of the effects of biogenic amines in consumers after the digestion of foods containing different levels of these compounds. Moreover, it is important to have quick, reliable, and precise analytical techniques to determine not only histamine and tyramine levels individually, but also to analyze other biogenic amines (putrescine, cadaverine, β-phenylethylamine, etc.) with implications for health and metabolic processes.

Such control of biogenic amines would benefit public authorities, industry, and consumers as it would help put higher quality products with fewer health implications on the market. However, guaranteeing the quality and safety of food requires a commitment not only from public institutions but also from production sectors, commercial processors, and ultimately from consumers who must play an important and active role in achieving food safety.

## 7. Future Trends and Perspectives

There are many lines of research looking into BAs in food and there are also many possibilities to be explored with regard to this subject from the technological, microbiological, analytical, and toxicological points of view.

Work should focus on determining the real risk of toxicity for consumers posed by BAs in food and should not be limited to a single amine or food product but should rather cover all the amines involved and in all foods consumed. Attention should also be given to the other factors contributing to toxicity, such as toxicity enhancers (individual susceptibility, consumption of MAOI, synergies resulting from the consumption of foods, etc.). Although these aspects are truly difficult to address, they should be studied and included in future regulations to guarantee food safety and consumer health.

Another important reason to control these compounds is the fact that often foods with toxic levels of BAs, such as histamine or tyramine, appear organoleptically ‘normal’ and consumers are unable to reject products based on sensorial parameters.

Moreover, today’s market is trending towards the development of new products with new ingredients and new processing technologies, which create new conditions that could either favor or reduce the formation of biogenic amines. This is the case, for example, of the effect of decarboxylase enzymes responsible for their formation and the factors that modulate this activity. Therefore the implications of these new factors must be taken into account in new projects.

Important research efforts should continue in the field of analysis and determination of these BAs, always focused on the simultaneous determination of all of them, and on the different matrices, in order to solve the problems of extraction and interference of complex matrices. Also, advances need to be made in the search for more accurate, swift, simple, and unified determination methods that can easily be transferred to laboratories, industry, and the public administration.

Consequently, all research efforts should focus on the overarching goal of food safety and on providing the authorities with the tools they need to conduct swift checks of these compounds to reduce risk to consumers.

## Figures and Tables

**Table 1:** Methods and conditions for the determination of biogenic amines in food samples.

Analyte	Method/ Equipment	Sample	Extraction Solvents	Separation Technique	Derivatization Reagents	Detection System	Time of Analysis (min)	LOD	Ref
HIS	Fluorometric	Seafood, meats, cheeses, sauerkraut, etc.	MeOH	---	OPA	PF	---	0.02 mg/100 g	[59]
HIS	Fluorometric	Fish (fresh, dry, salted, frozen, brine, etc.)	MeOH	Ion exchange resin	OPA	PF	---	--	[60]
HIS	Colorimetric	Fish (tuna, mackerel),	NaCl solution	---	p-phenyldiazonium sulfonate	UV/vis	---	1 mg/100 g	[61]
HIS	ELISA immunoassay	Fish, wine		---	---	UV/vis	10	--	[62]
HIS, Tyr, Cad, Put, Phe, Trp, Spd, Spm	TLC	Cod, squid, MRS, TSB	TCA	---	Dansyl chloride	PF	---	5 ng–10 ng (1 mg/L per 10 μL spotted)	[63,64]
HIS, Cad, Put, Spm	IEC	Tuna fish	MSA, HCL, PCA, PB	IonPac CS17	MSA	EDC	20	0.15–0.50 mg/kg	[65]
HIS, Tyr, Cad, Put, Spd	HPLC	Milk (cow, goat)	PCA	ODS2-C18	Benzoyl Chloride	UV/vis	13	0.03–1.30 mg/L	[66]
His, Tyr, Phe, Try, Cad, Put Spm Spd	HPLC	Sausages, cheese	PCA, HCL	Eclipse XDB-C18	Dansyl chloride, Fluorenylmethoxy-carbonyl chloride, Benzoyl chloride, Dansyl chloride	UV/vis	25–50	0.03–0.38 mg/kg,	[67]
HIS, Tyr, Cad, Put, Phe, Trp, Spd, Spm	HPLC or UHPLC	Meat, beer, wine, rice, mushroom, sausage, juice, oil, peanut butter, fish, shrimp sauce, etc.	PCA TCA HCL	ODS2-C-18, Nova-Pak C18, Zorbax XDB C18	Dansyl chloride	UV/vis	6–30	0.01–0.10 mg/kg 4.43–6.96 μg/L	[68,69,70,71]
HIS, Tyr, Cad, Put, Phe, Spd, Spm, Ser, Met, Etm	HPLC or UHPLC	Wines, meat, beverages, coffee	TCA	Phenomenex Luna 5u RP-18 Kromasil	Dansyl chloride	DAD	35	0.5 mg/kg	[72,73]
Trp, Phe, Put, Cad, HIS, Tyr, Spm, Spd	HPLC	Chicken carcasses	PCA	C18	Dansyl chloride	FLD	32	0.05–25 μg/mL	[74]
HIS, Met, Etm, Tyr, Phe, Put, Cad	HPLC	Wine	---	Nova-Pak C18	OPA	FLD	42	0.006–0.057 mg/L	[62]
HIS, Tyr, Cad, Put, Phe, Agm, Trp, Spd, Spm	HPLC or UHPLC	Meat, fish, squid, prawn	TCA PCA	Cation exchange-Capcell Pak MG-C18	OPA	FLD	25–55	0.05–0.2 mg/L 0.2–2.0 µg/L	[75,76,77]
Met, Etm, HIS, Tym, Trp, Phe, Put, Cad	HPLC	Canned tuna fish	TCA	Inertsil ODS-3	Naphthalene-2,3-dicarboxaldehyde	FLD	50	2.5–330 mg/kg	[78]
HIS, Tyr, Phe, Ser, Trp, Oct, Dopa, Cad, Put, Agm, Spd, Spm	HPLC	Wine, cider, spinach hazelnut, banana, potato, milk, chocolate, meat	PCA	Nova-Pak C18	OPA	FLD	55–60	0.03–0.06 mg/L 0.07–0.2 mg/L ≤1.5 mg/kg	[41,79,80]
HIS, Tyr, Phe, Ser, Trp, Oct, Dopa, Cad, Put, Agm, Spd, Spm	UHPLC	Wine, fish, cheese, sausage	PCA	Acquity BEH C18	OPA	FLD	7	0.2–0.3 mg/L	[81]
Put, HIS, Cad, Phe, Tyr, Spd, Spm	HPLC-	Beer, cheese, fish, sausage, shrimp	TCA	Hypersil BDS C18	EAC	FLD	6	0.27–0.69 ng/mL	[82]
HIS, Tyr, Cad, Put, Phe, Agm, Trp, Spd, Spm, Ser, Oct, Dopa	HPLC	Wines	---	A Zorbax C18	NQS	DAD	45	0.2–3 mg/L,	[83]
Met, HIS, Put, Cad, Tyr, Spm, Spd, Trip, Phe, Etm	HPLC	Wine, beer	PVP	Inertsil ODS-3 column	Dansyl chloride	DAD–APCI-MS	35	0.008–40.0 mg/L	[84]
His, Tyr, Spd, Spm, Cad, Put, Agm	HPLC	Fish, cheese, meat, vegetable	HCL, TCA, MeOH	LiChrospher RP 18,	OPA	FLD–DAD	20	0.5–8.5 mg/kg	[85]
HIS, Cad, Agm, Tyr, Put, Phe	HPLC	Beer, wines	BB	Gemini C-18	*p*-toluenesulfonyl chloride	MS	22	0.023–12 µg/dm^3^	[86]
HIS, Tyr, Phe	HPLC	Cheese	HCL	Luna C18	---	MS	11	0.05–0.25 mg/kg	[87]
Cad, Put, HIS	GC	Cheese, fish	---	OV-225	Perfluoropropionyl derivatives	ECD	20	<1.1 µg/g	[88]
Etm, HIS, Put, Spm, Trp, Tyr, Phe, Met, Prp	GC	Wine	MeOH, CHCl3 (DLLME)	ZB-5MS capillary column	IBCF, PCF	MS–MS	25	<4.1 μg/L.	[89]
Cad, Put HIS	GC	Apple juice	DLLME	CC-DB-5	---	MS	8	0.06–2.20 μg/L	[90]
Put, Cad, HIS, Phe, Tyr	GC	Alcoholic beverages	Toluene	CC-HP-5MS	Isobutyl chloroformate	MS	12	1–10 μg/L	[91]
HIS	FIA	Mackerel, mahi-mahi	MeOH	---	OPA	FLD	---	0.8–6 mg/kg	[92,93]
HIS	FIA	Cider, wine	---	Anion exchange mini-column	OPA	FLD	---	30–101 µg/L	[94]
HIS, Tyr, Put, Cad, Agm, Spm	FIA	Tuna	Water	Electrode-Biosensor (AO, HmDH)	OPA	APMD	---	100 pmol	[95,96]
HIS, Phe	CE–FIA	Standard solutions	Water	---	---	MS	22	0.018–0.09 μg/mL	[97]
Put, Cad, Spm, Spd, Trp, Tyr, HIS	CE	Sauerkraut	PCA	Silica capillary	Benzoyl chloride	UV/vis	35	0.2–0.7 mg/L	[98]
HIS, Tyr, Phe, Put, Cad, Spm, Spd	CE	Soy sauce, fish, wine	TCA	Silica capillary	FBQCA	LIFD	14	0.4–10 nM	[99]
Put, Cad, Spd, Spm	CE	Fresh milk	PCA	Ag/AgCl electrode	---	APMD	27	100–400 nM	[100]
Put, HIS, Try, Phe, Spd	CE	Oyster	PCA	capillary column- Ag/AgCl	---	ECHL	30	9.2 × 10^−4^–9.6 × 10^−2^ μg/mL	[101]
HIS, Tyr	CE	Meat, cheese, fish, vegetable	HCL, MeOH, TCA	---	---	DAD	9	2–6 mg/kg	[85]
Spm, Spd, Put, Cad, HIS, Phe, Trp, Tyr	CE	Beer, wine	---	Electrophoretic separation	---	MS	10	1–2 μg/L	[102]

Agm: agmatine, AO: amine oxidase, APMD: amperometric detection, BB: borate-buffer, Cad: cadaverine, CC: capillary column, CE: capillary electrophoresis, CHCl3: chloroform, CHMD: chemiluminescence detector, DAD: diode-array detector, DAD–APCI-MS: diode array detection–atmospheric pressure chemical ionization mass spectrometry system, DLLME: dispersive liquid microextraction, DOPA: dopamine, EAC: ethyl-acridine-sulfonyl chloride, EB: electrode-biosensor, ECD: electron capture detector, ECHL: electrochemiluminescence, EDC: electrochemical detector-conductivity, Etm: ethylamine, FBQCA: 3-(4-fluorobenzoyl)-2-quinolinecarboxaldehyde, FLD: fluorescence detection, HCL: chloridric acid, HIS: histamine, HmDH: histamine dehydrogenase, HPLC: high-performance liquid chromatography or high pressure liquid chromatography, HS-SPME: head space solid phase microextraction; IBCF: isobutyl choloroformate; IBUT: isobutylamine, IEC: ion-exchange chromatography, LIFD: laser-induced fluorescence detection, LLE: liquid-liquid extraction; LOD: limits of the detection, MAS: methanesulfonic acid, MeOH: methanol, Met: methylamine, MRS: Man, Rogosa and Sharpe Broth, MSA: methanesulphonic acid, MS: mass spectrometry, NQS: 1,2-naphthoquinone-4-sulfonate, Oct: octopamine, OPA: *o*-phthaldialdehyde, PB: phosphate buffer, PCA: percloric acid, PCF: propyl chloroformate, PF: photofluorometer, Phe: β-phenylethylamine, Put: putrescine, PVP: polyvinylpyrrolidone, Ser: serotonin, Spd: spermidine, Spm: spermine, TCA: tricloroacetic acid, TLC: thin layer chromatography, Trp: tryptamine, TSB: tryptic soy broth, Tyr: tyramine, UHPLC: ultra-high performance liquid chromatography, UV: ultraviolet.

## References

[1] FDA (Food and Drug Administration) Food Safety Modernization Act (FSMA). https://www.fda.gov/food/guidanceregulation/fsma/.

[2] CDCP (Centers for Disease Control and Prevention) https://www.cdc.gov/foodsafety/index.html.

[3] FDA (Food and Drug Administration) Fish and Fishery Products Hazards and Controls Guidance - Fourth Edition. https://www.fda.gov/Food/GuidanceRegulation/GuidanceDocumentsRegulatoryInformation/Seafood/ucm2018426.htm.

[4] EFSA (2011). Scientific Opinion on risk based control of biogenic amine formation in fermented foods. EFSA J..

[5] Bardócz S. (1995). Polyamines in food and their consequences for food quality and human health. Trends Food Sci. Technol..

[6] Kalač P. (2014). Health effects and occurrence of dietary polyamines: A review for the period 2005-mid 2013. Food Chem..

[7] Taylor S.L., Eitenmiller R.R. (1986). Histamine food poisoning: Toxicology and clinical aspects. Crit. Rev. Toxicol..

[8] Lehane L., Olley J. (2000). Histamine fish poisoning revisited. Int. J. Food Microbiol..

[9] Kim M.K., Mah J.H., Hwang H.J. (2009). Biogenic amine formation and bacterial contribution in fish, squid and shellfish. Food Chem..

[10] Pegg A.E. (2013). Toxicity of polyamines and their metabolic products. Chem. Res. Toxicol..

[11] Kovacova-Hanuskova E., Buday T., Gavliakova S., Plevkova J. (2015). Histamine, histamine intoxication and intolerance. Allergologia et Immunopathologia.

[12] Prester L. (2011). Biogenic amines in fish, fish products and shellfish: A review. Food Addit. Contam. Part A Chem. Anal. Control Expo Risk Assess..

[13] Halász A., Baráth Á., Simon-Sarkadi L., Holzapfel W. (1994). Biogenic amines and their production by microorganisms in food. Trends Food Sci. Technol..

[14] Ruiz-Capillas C., Jiménez-Colmenero F. (2004). Biogenic amines in meat and meat products. Crit. Rev. Food Sci. Nutr..

[15] Karovičová J., Kohajdová Z. (2005). Biogenic amines in food. Chem. Papers.

[16] Linares D.M., Martĺn M.C., Ladero V., Alvarez M.A., Fernández M. (2011). Biogenic amines in dairy products. Crit. Rev. Food Sci. Nutr..

[17] Benkerroum N. (2016). Biogenic Amines in Dairy Products: Origin, Incidence, and Control Means. Compr. Rev. Food Sci. Food Saf..

[18] Rice S.L., Eitenmiller R.R., Koehler P.E. (1976). Biologically active amines in food: A review. J. Milk Food Technol..

[19] Hernández-Jover T., Izquierdo-Pulido M., Veciana-Nogués M.T., Vidal-Carou M.C. (1996). Biogenic Amine Sources in Cooked Cured Shoulder Pork. J. Agric. Food Chem..

[20] Suzzi G., Gardini F. (2003). Biogenic amines in dry fermented sausages: A review. Int. J. Food Microbiol..

[21] Stadnik J., Dolatowski Z.J. (2010). Biogenic amines in meat and fermented meat products. Acta Sci. Pol. Technol. Aliment.

[22] Kalač P., Krausová P. (2005). A review of dietary polyamines: Formation, implications for growth and health and occurrence in foods. Food Chem..

[23] Shalaby A.R. (1996). Significance of biogenic amines to food safety and human health. Food Res. Int..

[24] Al Bulushi I., Poole S., Deeth H.C., Dykes G.A. (2009). Biogenic amines in fish: Roles in intoxication, spoilage, and nitrosamine formation-A review. Crit. Rev. Food Sci. Nutr..

[25] De Mey E., De Klerck K., De Maere H., Dewulf L., Derdelinckx G., Peeters M.C., Fraeye I., Vander Heyden Y., Paelinck H. (2014). The occurrence of N-nitrosamines, residual nitrite and biogenic amines in commercial dry fermented sausages and evaluation of their occasional relation. Meat Sci..

[26] Ruiz-Capillas C., Carballo J., Jiménez Colmenero F. (2007). Biogenic amines in pressurized vacuum-packaged cooked sliced ham under different chilled storage conditions. Meat Sci..

[27] Alvarez M.A., Moreno-Arribas M.V. (2014). The problem of biogenic amines in fermented foods and the use of potential biogenic amine-degrading microorganisms as a solution. Trends Food Sci. Technol..

[28] Vidal-Carou M.C., Izquierdo-Pulido M.L., Martín-Morro M.C., Mariné F. (1990). Histamine and tyramine in meat products: Relationship with meat spoilage. Food Chem..

[29] Santos C., Jalón M., Marine A. (1985). Contenido de tiramina en alimentos de origen animal. I. Carne, derivados cárnicos y productos relacionados. Rev. Agroquim Technol. Aliment..

[30] McCabe-Sellers B.J., Staggs C.G., Bogle M.L. (2006). Tyramine in foods and monoamine oxidase inhibitor drugs: A crossroad where medicine, nutrition, pharmacy, and food industry converge. J. Food Composit. Anal..

[31] Herrero A.M. (2008). Raman spectroscopy a promising technique for quality assessment of meat and fish: A review. Food Chem..

[32] Mietz J.L., Karmas E. (1978). Polyamine and histamine content of rockfish, salmon, lobster, and shrimp as an indicator of decomposition. J. Assoc. Off. Anal. Chem. (USA).

[33] Smith T.A. (1980). Amines in food. Food Chem..

[34] Ruiz-Capillas C., Moral A. (2001). Production of biogenic amines and their potential use as quality control indices for hake (*Merluccius merluccius*, L.) stored in ice. J. Food Sci..

[35] Rokka M., Eerola S., Smolander M., Alakomi H.-L., Ahvenainen R. (2004). Monitoring of the quality of modified atmosphere packaged broiler chicken cuts stored in different temperature conditions: B. Biogenic amines as quality-indicating metabolites. Food Control.

[36] Ruiz-Capillas C., Jiménez-Colmenero F., Leo M.L., Nollet F.T. (2009). Biogenic amines in seafood products. Handbook of Seafood and Seafood Products Analysis.

[37] Galgano F., Favati F., Bonadio M., Lorusso V., Romano P. (2009). Role of biogenic amines as index of freshness in beef meat packed with different biopolymeric materials. Food Res. Int..

[38] Vinci G., Antonelli M.L. (2002). Biogenic amines: Quality index of freshness in red and white meat. Food Control.

[39] Kalač P., Křížek M. (2003). A review of biogenic amines and polyamines in beer. J. Inst. Brewing.

[40] Triki M., Herrero A.M., Jiménez-Colmenero F., Ruiz-Capillas C. (2018). Quality Assessment of Fresh Meat from Several Species Based on Free Amino Acid and Biogenic Amine Contents during Chilled Storage. Foods.

[41] Hernández-Jover T., Izquierdo-Pulido M., Veciana-Nogués M.T., Vidal-Carou M.C. (1996). Ion-Pair High-Performance Liquid Chromatographic Determination of Biogenic Amines in Meat and Meat Products. J. Agric. Food Chem..

[42] Latorre-Moratalla M.L., Veciana-Nogués T., Bover-Cid S., Garriga M., Aymerich T., Zanardi E., Ianieri A., Fraqueza M.J., Patarata L., Drosinos E.H. (2008). Biogenic amines in traditional fermented sausages produced in selected European countries. Food Chem..

[43] Gardini F., Özogul Y., Suzzi G., Tabanelli G., Özogul F. (2016). Technological factors affecting biogenic amine content in foods: A review. Frontiers in Microbiology.

[44] Eerola H.S., Roig Sagués A.X., Hirvi T.K. (1998). Biogenic amines in Finnish dry sausages. J. Food Saf..

[45] Silla Santos M.H. (1996). Biogenic amines: Their importance in foods. Int. J. Food Microbiol..

[46] Bodmer S., Imark C., Kneubühl M. (1999). Biogenic amines in foods: Histamine and food processing. Inflamm. Res..

[47] Komprda T., Smělá D., Pechová P., Kalhotka L., Štencl J., Klejdus B. (2004). Effect of starter culture, spice mix and storage time and temperature on biogenic amine content of dry fermented sausages. Meat Sci..

[48] Roig-Roig-Sagués A.X., Ruiz-Capillas C., Espinosa D., Hernández M., Dandrifosse G. (2009). The decarboxylating bacteria present in foodstuffs and the effect of emerging technologies on their formation. Biological Aspects of Biogenic Amines, Polyamines and Conjugates.

[49] Naila A., Flint S., Fletcher G., Bremer P., Meerdink G. (2010). Control of biogenic amines in food - existing and emerging approaches. J. Food Sci..

[50] Kim J.H., Ahn H.J., Lee J.W., Park H.J., Ryu G.H., Kang I.J., Byun M.W. (2005). Effects of gamma irradiation on the biogenic amines in pepperoni with different packaging conditions. Food Chem..

[51] Ten Brink B., Damink C., Joosten H.M., Huis in ‘t Veld J.H. (1990). Occurrence and formation of biologically active amines in foods. Int. J. Food Microbiol..

[52] Bover-Cid S., Miguélez-Arrizado M.J., Vidal-Carou M.C. (2001). Biogenic amine accumulation in ripened sausages affected by the addition of sodium sulphite. Meat Sci..

[53] Ruiz-Capillas C., Herrero A.M., Jiménez-Colmenero F., Ruiz-Capillas C., Nollet L.M.L. (2015). Determination of biogenic amines. Flow Injection Analysis of Food Additives.

[54] Rivoira L., Zorz M., Martelanc M., Budal S., Carena D., Franko M., Bruzzoniti M.C. (2017). Novel approaches for the determination of biogenic amines in food samples. Stud. u. Babes-Bol. Chem..

[55] Önal A. (2007). A review: Current analytical methods for the determination of biogenic amines in foods. Food Chem..

[56] Mohammed G.I., Bashammakh A.S., Alsibaai A.A., Alwael H., El-Shahawi M.S. (2016). A critical overview on the chemistry, clean-up and recent advances in analysis of biogenic amines in foodstuffs. Trends Anal. Chem..

[57] Ordóñez J.L., Troncoso A.M., García-Parrilla M.D.C., Callejón R.M. (2016). Recent trends in the determination of biogenic amines in fermented beverages–A review. Anal. Chim. Acta.

[58] Papageorgiou M., Lambropoulou D., Morrison C., Kłodzińska E., Namieśnik J., Płotka-Wasylka J. (2018). Literature update of analytical methods for biogenic amines determination in food and beverages. Trends Anal. Chem..

[59] Taylor S.L., Lieber E.R., Leatherwood M. (1978). A simplified method for histamine analysis of foods. J. Food Sci..

[60] Cunniff P.A., AOAC (1995). Histamine in seafood: Fluorometric method Sec. 35.1.32, Method 977.13. Official Methods of Analysis of AOAC International.

[61] Patange S.B., Mukundan M.K., Kumar K.A. (2005). A simple and rapid method for colorimetric determination of histamine in fish flesh. Food Control.

[62] Marcobal A., Polo M.C., Martín-Álvarez P.J., Moreno-Arribas M.V. (2005). Biogenic amine content of red Spanish wines: Comparison of a direct ELISA and an HPLC method for the determination of histamine in wines. Food Res. Int..

[63] Lapa-Guimarães J., Pickova J. (2004). New solvent systems for thin-layer chromatographic determination of nine biogenic amines in fish and squid. J. Chromatogr..

[64] Latorre-Moratalla M.L., Bover-Cid S., Veciana-Nogués T., Vidal-Carou M.C. (2009). Thin-layer chromatography for the identification and semi-quantification of biogenic amines produced by bacteria. J. Chromatogr..

[65] Cinquina A.L., Calì A., Longo F., De Santis L., Severoni A., Abballe F. (2004). Determination of biogenic amines in fish tissues by ion-exchange chromatography with conductivity detection. J. Chromatogr..

[66] Costa M.P., Balthazar C.F., Rodrigues B.L., Lazaro C.A., Silva A.C.O., Cruz A.G., Conte Junior C.A. (2015). Determination of biogenic amines by high-performance liquid chromatography (HPLC-DAD) in probiotic cow’s and goat’s fermented milks and acceptance. Food Sci. Nutr..

[67] Liu S.J., Xu J.J., Ma C.L., Guo C.F. (2018). A comparative analysis of derivatization strategies for the determination of biogenic amines in sausage and cheese by HPLC. Food Chem..

[68] Eerola S., Hinkkanen R., Lindfors E., Hirvi T. (1993). Liquid chromatographic determination of biogenic amines in dry sausages. J. AOAC Int..

[69] Yoon H., Park J.H., Choi A., Hwang H.J., Mah J.H. (2015). Validation of an HPLC analytical method for determination of biogenic amines in agricultural products and monitoring of biogenic amines in Korean fermented agricultural products. Toxicol. Res..

[70] Dadáková E., Křížek M., Pelikánová T. (2009). Determination of biogenic amines in foods using ultra-performance liquid chromatography (UPLC). Food Chem..

[71] Saaid M., Saad B., Hashim N.H., Mohamed Ali A.S., Saleh M.I. (2009). Determination of biogenic amines in selected Malaysian food. Food Chem..

[72] Anli R.E., Vural N., Yilmaz S., Vural Ỳ.H. (2004). The determination of biogenic amines in Turkish red wines. J. Food Compos. Anal..

[73] Casal S., Oliveira M.B.P.P., Ferreira M.A. (2002). Determination of biogenic amines in coffee by an optimized liquid chromatographic method. J. Liq. Chromatogr. Relat. Technol..

[74] Tamim N.M., Bennett L.W., Shellem T.A., Doerr J.A. (2002). High-performance liquid chromatographic determination of biogenic amines in poultry carcasses. J. Agric. Food Chem..

[75] Triki M., Jiménez-Colmenero F., Herrero A.M., Ruiz-Capillas C. (2012). Optimisation of a chromatographic procedure for determining biogenic amine concentrations in meat and meat products employing a cation-exchange column with a post-column system. Food Chem..

[76] Sánchez J.A., Ruiz-Capillas C. (2012). Application of the simplex method for optimization of chromatographic analysis of biogenic amines in fish. Eur. Food Res. Technol..

[77] Zhao Q.X., Xu J., Xue C.H., Sheng W.J., Gao R.C., Xue Y., Li Z.J. (2007). Determination of biogenic amines in squid and white prawn by high-performance liquid chromatography with postcolumn derivatization. J. Agric. Food Chem..

[78] Zotou A., Notou M. (2013). Enhancing Fluorescence LC Analysis of Biogenic Amines in Fish Tissues by Precolumn Derivatization with Naphthalene-2,3-dicarboxaldehyde. Food Anal. Method..

[79] Vidal-Carou M.C., Lahoz-Portolés F., Bover-Cid S., Mariné-Font A. (2003). Ion-pair high-performance liquid chromatographic determination of biogenic amines and polyamines in wine and other alcoholic beverages. J. Chromatogr..

[80] Lavizzari T., Teresa Veciana-Nogués M., Bover-Cid S., Mariné-Font A., Carmen Vidal-Carou M. (2006). Improved method for the determination of biogenic amines and polyamines in vegetable products by ion-pair high-performance liquid chromatography. J. Chromatogr..

[81] Latorre-Moratalla M.L., Bosch-Fusté J., Lavizzari T., Bover-Cid S., Veciana-Nogués M.T., Vidal-Carou M.C. (2009). Validation of an ultra high pressure liquid chromatographic method for the determination of biologically active amines in food. J. Chromatogr..

[82] Li G., Dong L., Wang A., Wang W., Hu N., You J. (2014). Simultaneous determination of biogenic amines and estrogens in foodstuff by an improved HPLC method combining with fluorescence labeling. LWT Food Sci. Technol..

[83] Hlabangana L., Hernández-Cassou S., Saurina J. (2006). Determination of biogenic amines in wines by ion-pair liquid chromatography and post-column derivatization with 1,2-naphthoquinone-4-sulphonate. J. Chromatogr..

[84] Loukou Z., Zotou A. (2003). Determination of biogenic amines as dansyl derivatives in alcoholic beverages by high-performance liquid chromatography with fluorimetric detection and characterization of the dansylated amines by liquid chromatography-atmospheric pressure chemical ionization mass spectrometry. J. Chromatogr..

[85] Lange J., Thomas K., Wittmann C. (2002). Comparison of a capillary electrophoresis method with high-performance liquid chromatography for the determination of biogenic amines in various food samples. J. Chromatogr. B Analyt. Technol. Biomed. Life Sci..

[86] Nalazek-Rudnicka K., Wasik A. (2017). Development and validation of an LC–MS/MS method for the determination of biogenic amines in wines and beers. Monatshefte fur Chemie.

[87] Calbiani F., Careri M., Elviri L., Mangia A., Pistarà L., Zagnoni I. (2005). Rapid assay for analyzing biogenic amines in cheese: Matrix solid-phase dispersion followed by liquid chromatography-electrospray-tandem mass spectrometry. J. Agric. Food Chem..

[88] Staruszkiewicz W.F., Bond J.F. (1981). Gas chromatographic determination of cadaverine, putrescine, and histamine in foods. J. Assoc. Off. Anal. Chem..

[89] Płotka-Wasylka J., Simeonov V., Namieśnik J. (2016). An in situ derivatization - dispersive liquid-liquid microextraction combined with gas-chromatography - mass spectrometry for determining biogenic amines in home-made fermented alcoholic drinks. J. Chromatogr..

[90] Cunha S.C., Faria M.A., Fernandes J.O. (2011). Gas chromatography-mass spectrometry assessment of amines in port wine and grape juice after fast chloroformate extraction/derivatization. J. Agric. Food Chem..

[91] Fernandes J.O., Judas I.C., Oliveira M.B., Ferreira I.M.P.L.V., Ferreira M.A. (2001). A GC-MS method for quantitation of histamine and other biogenic amines in beer. Chromatographia.

[92] Hungerford J.M., Walker K.D., Wekell M.M., LaRose J.E., Throm H.R. (1990). Selective Determination of Histamine by Flow Injection Analysis. Anal. Chem..

[93] Hungerford J.M., Hollingworth T.A., Wekell M.M. (2001). Automated kinetics-enhanced flow-injection method for histamine in regulatory laboratories: Rapid screening and suitability requirements. Anal. Chim. Acta.

[94] Del Campo G., Gallego B., Berregi I. (2006). Fluorimetric determination of histamine in wine and cider by using an anion-exchange column-FIA system and factorial design study. Talanta.

[95] Niculescu M., Frébort I., Peč P., Galuszka P., Mattiasson B., Csöregi E. (2000). Amine oxidase based amperometric biosensors for histamine detection. Electroanalysis.

[96] Takagi K., Shikata S. (2004). Flow injection determination of histamine with a histamine dehydrogenase-based electrode. Anal. Chim. Acta.

[97] Santos B., Simonet B.M., Ríos A., Valcárcel M. (2004). Direct automatic determination of biogenic amines in wine by flow injection-capillary electrophoresis-mass spectrometry. Electrophoresis.

[98] Křížek M., Pelikánová T. (1998). Determination of seven biogenic amines in foods by micellar electrokinetic capillary chromatography. J. Chromatogr..

[99] Zhang N., Wang H., Zhang Z.X., Deng Y.H., Zhang H.S. (2008). Sensitive determination of biogenic amines by capillary electrophoresis with a new fluorogenic reagent 3-(4-fluorobenzoyl)-2-quinolinecarboxaldehyde. Talanta.

[100] Sun X., Yang X., Wang E. (2003). Determination of biogenic amines by capillary electrophoresis with pulsed amperometric detection. J. Chromatogr..

[101] An D., Chen Z., Zheng J., Chen S., Wang L., Huang Z., Weng L. (2015). Determination of biogenic amines in oysters by capillary electrophoresis coupled with electrochemiluminescence. Food Chem..

[102] Daniel D., dos Santos V.B., Vidal D.T.R., do Lago C.L. (2015). Determination of biogenic amines in beer and wine by capillary electrophoresis-tandem mass spectrometry. J. Chromatogr..

